# Plasma proteomics identifies molecular subtypes in sepsis

**DOI:** 10.1186/s13054-025-05639-6

**Published:** 2025-09-02

**Authors:** Thilo Bracht, Kerstin Kappler, Malte Bayer, Franziska Grell, Karin Schork, Lars Palmowski, Björn Koos, Tim Rahmel, Dominik Ziehe, Matthias Unterberg, Lars Bergmann, Katharina Rump, Martina Broecker-Preuss, Ulrich Limper, Dietrich Henzler, Stefan Felix Ehrentraut, Thilo von Groote, Alexander Zarbock, Stephanie Pfaender, Nina Babel, Katrin Marcus-Alic, Martin Eisenacher, Michael Adamzik, Barbara Sitek, Hartmuth Nowak

**Affiliations:** 1 Knappschaft Kliniken Universitätsklinikum Bochum, Klinik für Anästhesiologie, Intensivmedizin und Schmerztherapie, Ruhr University Bochum, Bochum, Germany; 2 Medical Faculty, Medizinisches Proteom-Center, Ruhr University Bochum, Bochum, Germany; 3 Knappschaft Kliniken Universitätsklinikum Bochum, Center for Artificial Intelligence, Medical Informatics and Data Science, Ruhr University Bochum, Bochum, Germany; 4 Medical Faculty, Center for Protein Diagnostics (PRODI), Medical Proteome Analysis, Ruhr-University Bochum, Bochum, Germany; 5 CUBiMed.RUB, Core Unit Bioinformatics, Medical Faculty, Ruhr University Bochum, Bochum, Germany; 6 University Hospital Knappschaftskrankenhaus, Department of Internal Medicine, Laboratory Medicine Section, Ruhr University Bochum, 44892 Bochum, Germany; 7 Department of Anesthesiology and Operative Intensive Care Medicine, University of Witten/Herdecke, Cologne Merheim Medical School, 51109 Cologne, Germany; 8 Klinikum Herford, Department of Anesthesiology, Surgical Intensive Care, Emergency and Pain Medicine, Ruhr University Bochum, 32049 Herford, Germany; 9 Klinik für Anästhesiologie und Operative Intensivmedizin, Universitätsklinikum Bonn, 53127 Bonn, Germany; 10 Department of Anesthesiology, Intensive Care and Pain Medicine, University Hospital of Münster, Münster, Germany; 11 Research Unit: Emerging Viruses, Leibniz Institute of Virology (N63), 20251 Hamburg, Germany; 12 Institute of Virology and Cell Biology, University of Lübeck, Lübeck, Germany; 13 Centrum für Translationale Medizin mit Schwerpunkt Immunologie und Transplantation, Marien Hospital Herne, Universitätsklinikum der Ruhr-Universität Bochum, Herne, Germany

**Keywords:** Sepsis, Subclasses, Plasma, Clinical routine data, Machine learning, Hierarchical clustering, Precision medicine

## Abstract

**Background:**

The heterogeneity of sepsis represents a significant challenge to the development of personalized sepsis therapies. Sepsis subtyping has therefore emerged as an important approach to this problem, but its impact on clinical practice was limited due to insufficient molecular insights. Modern proteomics techniques allow the identification of subtypes and provide molecular and mechanistical insights. In this study, we analyzed a prospective multi-center sepsis cohort using plasma proteomics to describe and characterize sepsis plasma proteome subtypes.

**Methods:**

Plasma samples were collected from 333 patients at days 1 and 4 of sepsis and analyzed using liquid chromatography coupled to tandem mass spectrometry. Plasma proteome subtypes were identified using K-means clustering and characterized based on clinical routine data, cytokine measurements, and proteomics data. A random forest machine learning classifier was generated to showcase future assignment of patients to subtypes.

**Results:**

Four subtypes with different sepsis severity were identified. Cluster 0 represented the most severe form of sepsis, with 100% mortality. Cluster 1, 2 and 3 showed a gradual decrease of the median SOFA score, as reflected by clinical data and cytokine measurements. At the proteome level, the subtypes were characterized by distinct molecular features. We observed an alternating immune response, with cluster 1 showing prominent activation of the adaptive immune system, as indicated by elevated levels immunoglobulin (Ig) levels, which were verified using orthogonal measurements. Cluster 2 was characterized by acute inflammation and the lowest Ig levels. Cluster 3 represented the sepsis proteome baseline of the investigated cohort. We generated an ML classifier and optimized it for the minimum number of proteins that could realistically be implemented into routine diagnostics. The model, which was based on 10 proteins and Ig quantities, allowed the assignment of patients to clusters 1, 2 and 3 with high confidence.

**Conclusion:**

The identified plasma proteome subtypes provide insights into the immune response and disease mechanisms and allow conclusions on appropriate therapeutic measures, enabling predictive enrichment in clinical trials. Thus, they represent a step forward in the development of targeted therapies and personalized medicine for sepsis.

**Supplementary Information:**

The online version contains supplementary material available at 10.1186/s13054-025-05639-6.

## Introduction

Sepsis is a life-threatening condition caused by a dysregulated immune response to an infection [1]. It is a frequent cause of death in intensive care units (ICU) and represents a major burden in global healthcare with persistently high mortality rates [2]. Up to now, the major improvements in sepsis therapy were based on the effective treatment of the underlying infection and supportive measures [3]. All therapeutic approaches that specifically targeted molecular mechanisms in sepsis failed in large clinical trials [4]. The major reason for this is seen in the fact that sepsis is a complex, multifactorial syndrome and patient subgroups that might benefit from the tested medication were masked by others in heterogeneous clinical cohorts [5]. The concept of personalized medicine is based on the identification and treatment of only those patients that benefit from a therapy. This, however, requires accurate knowledge of the pathophysiological processes underlying the disease and appropriate markers for patient stratification.

In this respect, high throughput technologies can be used to screen patients for biomarkers and elucidate molecular disease mechanisms in an unbiased manner. Mass spectrometry-based plasma proteomics have emerged as a powerful analytical tool, giving access to large patient cohorts and a wide range of blood proteins. Despite these opportunities, studies that analyzed the blood proteome of septic patients remain comparably rare [6–9]. A landmark study published in 2024 was the first with a cohort size that allowed the identification of patient subgroups representing molecular sepsis subtypes [10]. This approach was complementary to others, that mostly used clinical standard parameters or transcriptomic data [11–14]. The generalizability of phenotyping studies, however, remains questionable, especially between different geographic locations [15, 16]. We conclude that beyond the mere identification, the molecular and clinical characterization of subtypes is an absolute necessity to receive transferable insights. In addition, a deep characterization is crucial to elucidate the pathophysiological relevance of the observed subtypes and infer future therapeutic concepts.

In this study, we analyzed proteomics data from a multicenter cohort comprising 333 patients and samples collected at two time points in early sepsis. We applied cluster analysis and identified four sepsis plasma proteome subtypes and their trajectories between both time points. We present a deep characterization of the subtypes and their clinical characteristics. In addition, we developed a supervised machine learning model to allocate new patients to the proteome clusters based on a minimized number of features, demonstrating how proteome subtypes could be implemented in clinical practice in the future.

## Methods

### Patient cohort

Patients with sepsis according to Sepsis-3 definition [1] were enrolled within the multi-center, prospective, observational SepsisDataNet.NRW and CovidDataNet.NRW studies (German Clinical Trial Registry, No. DRKS00018871). The diagnosis of sepsis had to be made within 48 h before study inclusion, which then was on the first day of treatment in the ICU. Blood samples (EDTA plasma and serum) were collected at day 1 and day 4. Patient-related ICU data and baseline characteristics were derived from the electronic patient’s records. Treatment was provided in accordance with current guidelines and was not influenced by study inclusion. No patients with limits on resuscitation or withdrawn supportive care were enrolled.

### Plasma proteomics

Proteome analyses of plasma samples were conducted as described recently [6]. Briefly, 1 µl of plasma per sample was digested using the SP3 protocol and 545 samples from 333 patients were analyzed distributed over eight batches. Batches C3 and S9 were analyzed on a Vanquish Neo UHPLC coupled to an Orbitrap Exploris 240. Batch S3 was analyzed using an Ultimate 3000 RSLCnano HPLC coupled to an Orbitrap Fusion Lumos mass spectrometer. All other batches were measured on an Ultimate 3000 RSLCnano HPLC coupled to an Orbitrap Exploris 240 (all Thermo Scientific, Bremen, Germany). The samples were measured in data-independent acquisition mode and data were processed using DIA-NN (v.1.8.1) searching the UniProt/SwissProt database restricted to *Homo sapiens* (v.2022_05). All batches were processed separately and subsequently normalized and evaluated as described before [17] (Supplementary Fig. 1). Functional annotation and enrichment analyses were performed using the STRING web interface (string db.org, v.12.0).

### Proteome clustering

Clustering was performed using day 1 protein intensities. Only proteins measured at both timepoints (days 1 and 4) with less than 30% missing values at each timepoint were included in the analysis. Correlating features remained in the data set (Supplementary Table 1). For clustering, missing values were imputed with the median. First, day 1 protein intensities were standardized using z-transformation for each protein. The standardization parameters (mean and standard deviation) were then applied to day 4 data to ensure consistent transformation across the timepoints. Subsequently, a Principal Component Analysis (PCA) was calculated for day 1 to reduce dimensionality. The resulting principal components were used to project day 4 data into the same reduced feature space. Sepsis plasma proteome subtypes were identified using the k-means algorithm on the PCA results of the first 35 components (> 70% of variance, Supplementary Table 2, Supplementary Figs. 2 and 3). The optimal number of clusters was determined using silhouette curves, Gap statistics, the Davies-Bouldin index and initial biological interpretation of the results, resulting in four distinct clusters (Supplementary Figs. 4–6). To assign day 4 data to the clusters, the Euclidean distance to each cluster centre was calculated, and each data point was assigned to the cluster with the nearest cluster centre. All calculations were performed using Python (v.3.10.14) and the packages pandas (v.2.1.1) and scikit-learn (v.1.3.1).

### Cytokine measurements

The LegendPlex Human Inflammation Panel 1 (Biolegend, San Diego) was used according to the manufacturer’s instructions as described previously [18]. Briefly, serum samples were mixed and incubated with the LegendPlex beads, subsequently washed, and incubated with detection antibodies. After a final washing step, the measurements were carried out using a flow cytometer (Canto II, BD Biosciences, CA). Cytokine concentrations were interpolated from calibration curves that were generated and measured together with the analyzed samples. Calculated concentrations below the lower limit of quantification (LOD) were considered to be zero, concentrations higher than the upper LOD were replaced by the respective upper LOD.

### Immunoglobulin quantification

Serum aliquots were thawed and centrifuged at 10000xg for 5 s to pellet precipitates. The supernatant was used to determine the concentration of IgG, IgA and IgM. The measurement was performed turbidimetrically on the Cobas c501 system (Roche Diagnostics, Mannheim, Germany) using Roche reagents (Tina-quant IgA, IgG and IgM, second gen.), according to the manufacturer’s instructions.

### Statistics

Clinical variables with multiple observations per day were aggregated by the median, minimum or maximum using the most clinical informative value (e.g. minimum value for quick value and maximum value for creatinine). The 25th percentile, median, and 75th percentile were calculated for each variable and subtype. For categorical variables, p-values were calculated using the chi-squared or fisher test and adjusted using Bonferroni correction. Kruskal-Wallis followed by Dunn’s post-hoc test for pairwise comparisons was applied for continuous variables. Differences in protein intensities between the four proteome subtypes were tested for statistical significance using ANOVA followed by Tukey’s post-hoc test. ANOVA p-values were corrected according to Benjamini-Hochberg. Relative changes between the clusters were calculated as ratios of mean protein intensities. The significance threshold was set as an ANOVA p_FDR_ value ≤ 0.05, a posthoc-test p value ≤ 0.05 and a ratio of means (RoM) ≥ 1.5 or ≤ 0.67.

### Machine learning

The proteomics data set was filtered for completeness as described above. In favour of quantities determined by clinical routine diagnostics, all entries representing immunoglobulins were removed and replaced by IgG, IgA, and IgM concentrations that were determined orthogonally. Proteins represented by routine measurements such as C-reactive protein (CRP*)*, Fibrinogen gamma chain (FGG*)*, Fibrinogen alpha chain (FGA), Fibrinogen beta chain (FGB), Hemoglobin subunit alpha (HBA2,) Hemoglobin subunit beta (HBB), and Hemoglobin subunit delta (HBD) were excluded as well (Supplementary Table 3). For the clinical dataset, routine ICU data with less than 30% missing values was considered. Variables with multiple observations per day were aggregated as described above and missing values were completed using data from the subsequent two days. Before model development, correlated features were removed by calculating the Pearson correlation between features and excluding one of the two features with an absolute correlation greater than 0.7 (Supplementary Table 4). For clinical data, only variables with a Kruskal–Wallis test *p*-value < 0.05 were used (Supplementary Tables 5 and 6). Cluster 0 was not considered for ML due to its low patient count, which was insufficient for robust modelling.

To identify the most relevant features, a multiclass classifier based on a Random Forest model was employed (Supplementary Fig. 7). The data were split into training and test sets (80% training, 20% test), and a 100 times Monte Carlo cross-validation (MCCV) was performed to evaluate the model metrics for different feature sets. Feature selection was conducted in a nested iterative manner: medical and proteomic features were added separately in two distinct loops. In the outer loop, medical features were incrementally included based on their importance ranking, while in the inner loop, proteomic features were iteratively added for each medical feature subset. This approach enabled independent evaluation of features from each domain. The order of features for iterative training was determined based on an additional 100 times MCCV (80% training, 20% validation). In this process, the median ranks of feature importance were computed using SHAP values (Shapley Additive Explanations) for medical and proteomics data. Missing values were imputed using the median in each MCCV iteration. The point at which the recall growth rate markedly declined was identified using the *KneeLocator* function of the keed package in Python. Based on this threshold, the most frequently selected proteins across MCCV iterations and IgA, IgG and IgM were used to train a Random Forest model (Fig. 3b), for which SHAP values were calculated to assess the contribution of each feature to the model’s predictions. The applied hyperparameters can be found in Supplementary Table 7. Calculations were done using Python (v.3.10.12) and the packages pandas (v.2.2.2), numpy (v.1.26.4), keed (v.0.8.5), shap (v0.46.0) and scikit-learn (v.1.5.1).

## Results

### Cohort characteristics

The cohort comprised *n* = 333 patients that were enrolled in five university hospitals in Germany. The median age was 64 years (IQR 55–75) with 117 females (37.9%) and 216 males (62.1%). 74 patients (24.3%) showed a SARS-CoV-2 infection and the median SOFA score was 9 (IQR 5–12). The 30-day mortality was 39.6% (*n* = 132 patients). None of the patients had preexisting acute or chronic liver failure. Number of patients with a preexisting chronic kidney disease or the need for renal replacement therapy were *n* = 69 (21%) and *n* = 15 (5%), respectively. For all patients, plasma proteome analyses were done for day 1 while measurements for day 4 plasma samples were available for 213 patients.

### Identification, trajectories and clinical representation of the sepsis plasma proteome subtypes

**Fig. 1:**
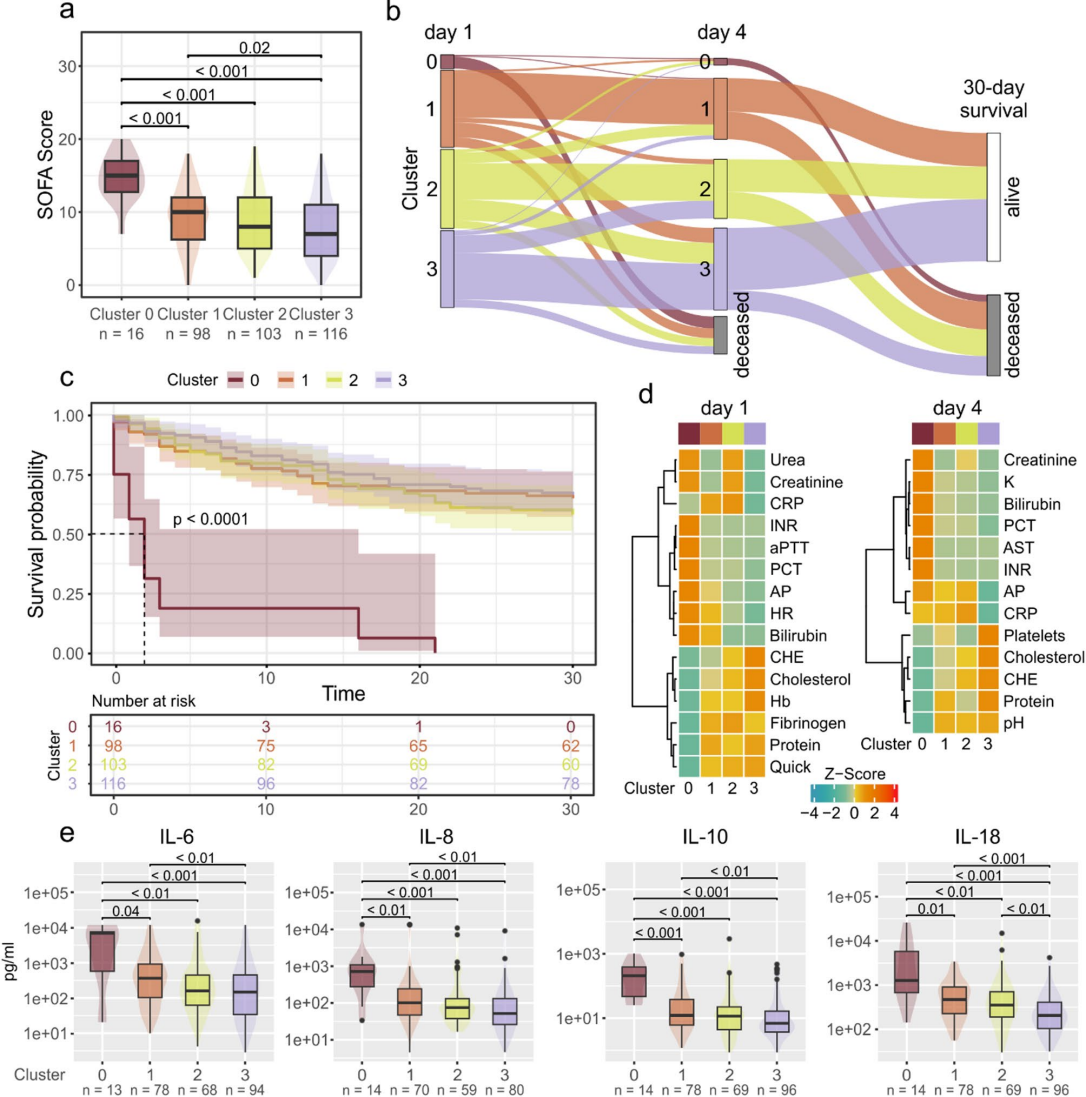
Clinical characteristics of sepsis plasma proteome subtypes. **a** Distribution of SOFA scores across the four clusters shown as boxplots. Boxes represent 25th and 75th percentiles, whiskers extend to the most extreme data points, median shown as a horizontal line, p-values from Dunn’s post-hoc test. **b** Sankey diagram showing the assignment of patients to proteome subtypes at day 1 and day 4, respectively. The endpoint is the 30-day survival. Data shown for *n* = 250 patients; *n* = 83 patients who were discharged from the ICU between day 1 and day 4 are not displayed. **c** Kaplan-Meier analysis according to 30-day survival for the four subtypes. **d** Heatmaps illustrating clinical routine data at day 1 and day 4. Only parameters which were significant between the clusters 1, 2 or 3 are shown (Bonferroni corrected Kruskal-Wallis followed by Dunn’s test). Data was aggregated by the mean, z-transformed, and clustered using Euclidean distance with Ward’s linkage method. **e** Boxplots illustrating cytokine measurements at day 1. Boxes represent 25th and 75th percentiles, whiskers extend to the most extreme data points, median shown as a horizontal line, outliers shown as individual data points, p-values from Dunn’s post-hoc test

The k-means algorithm identified four sepsis plasma proteome subtypes on data from day 1. The four clusters showed a decreasing severity of sepsis as represented by the SOFA score with the highest scores found in cluster 0 and the lowest in cluster 3 (Fig. 1a; Table 1). Consequently, the highest 30-day mortality was also observed in cluster 0 (Figs. 1b and c), while no significant differences in 30-day mortality were found between the other three clusters. The median survival time in cluster 0 was 2 days at day 1 (IQR 0–3) and 4 days at day 4 (IQR 3–4.5). No significant differences were found for age, sex and the focus of infection. Proteome data corresponding to day 4 were used to assign the respective patients to the same clusters. The results illustrate a relative stability of the clusters with the majority of patients staying in one cluster. Some patients, however, also migrated from one cluster to another. Migration to cluster 0 showed a striking relationship with the outcome, with all patients passing through cluster 0 eventually dying (Fig. 1b). Patients in cluster 0 showed significantly shorter length of the ICU and hospital stay (Supplementary Fig. 8). Of note, we observed a significant difference in 30-day mortality between clusters 2 and 3 at day 4, accompanied by a significantly shorter survival-time of patients in cluster 2 (Supplementary Fig. 9, Supplementary Table 5).

Clinically, cluster 0 was characterized by acute liver failure (AST, ALT, LDH), lactate acidosis and accompanying high-grade acute kidney injury (AKI). Furthermore, aPPT, INR, platelet count and fibrinogen concentration indicated disseminated intravascular coagulation (DIC). The decrease of CRP due to excessive consumption was less pronounced on day 4 compared to day 1 (Fig. 1d, Supplementary Table 5, Supplementary Fig. 10). These characteristics underlined the moribund character of patients in cluster 0. Cluster 1 showed a higher heart rate and elevated liver function markers AP, bilirubin, as well as lower CHE and cholesterol at day (1) On day 4, the heart rate was not significantly different between the four clusters, which was likely an effect of stabilization by the applied therapy. On day 1, cluster 2 was characterized by a moderate AKI with higher creatinine and urea levels and had the highest CRP levels of all four subtypes. Total protein was lower compared to clusters 1 and 3, an observation that was more pronounced on day 4, when cluster 2 also showed elevated AP levels and a decreased platelet count. Cluster 3 showed the overall mildest sepsis phenotypes and clinical values that were least alarming. Thus, it may be considered a baseline in our data set, however, at day 1 the 30-day survival was not significantly different from clusters 1 and 2. None of the three dysfunctions identified as most impactful - acute kidney injury, acute liver failure, and acute coagulopathy - were present prior to the onset of sepsis.

**Table 1:** Baseline characteristics at day of sepsis diagnose, differentiated by proteome clusters

	Total	Cluster 0 *n* = 16	Cluster 1 *n* = 98	Cluster 2 *n* = 103	Cluster 3 *n* = 116	*p* value	post-hoc testing					
							*p* (0 vs. 1)	*p* (0 vs. 2)	*p* (0 vs. 3)	*p* (1 vs. 2)	*p* (1 vs. 3)	*p* (2 vs. 3)
Age [years]	64 (55–75)	69 (64–77)	64 (55–72)	66 (56–77)	63 (52–74)	0.199	-	-	-	-	-	-
Female sex (%)	117 (38)	5 (31)	24 (28)	42 (44)	46 (42)	0.092	-	-	-	-	-	-
Focus of infection (%)						0.085	-	-	-	-	-	-
- intra-abdominal	71 (23)	8 (57)	25 (28)	23 (24)	15 (14)							
- bloodstream	15 (5)	1 (7)	6 (7)	6 (6)	2 (2)							
- urinary tract	19 (6)	0 (0)	6 (7)	8 (8)	5 (5)							
- pneumonia	103 (34)	3 (21)	28 (32)	30 (31)	42 (40)							
- CNS	9 (3)	0 (0)	1 (1)	3 (3)	5 (5)							
- COVID-19	74 (24)	1 (7)	17 (19))	26 (27)	30 (29)							
- other	13 (4)	1 (7)	5 (6)	2 (2)	5 (5)							
SOFA score (study inclusion)	9 (5–12)	15 (13–17)	10 (6–12)	8 (5–12)	7 (4–11)	< 0.001	< 0.001	< 0.001	< 0.001	0.310	0.015	0.150
Septic shock (%)	131 (47)	13 (100)	44 (54)	37 (40)	37 (40)	< 0.001	0.028	0.001	0.001	0.532	0.532	1.000
30-days mortality (%)	132 (40)	16 (100)	34 (35)	43 (42)	39 (34)	< 0.001	< 0.001	< 0.001	< 0.001	1.000	1.000	1.000
- Survival time^1^ [d]	8 (3–14)	2 (0–3)	7 (3–18)	12 (4–16)	9 (5–15)	< 0.001	< 0.001	< 0.001	< 0.001	0.970	0.970	0.804
**Inflammation**												
C-reactive protein [mg/dL]	16 (9–26)	14 (11–19)	20 (12–31)	21 (12–31)	11 (7–18)	< 0.001	0.831	0.578	0.831	0.831	< 0.001	< 0.001
WBC [x10^3^/µL]	12 (8–18)	15 (8–19)	13 (8–19)	14 (9–18)	11 (7–16)	0.213	-	-	-	-	-	-
Procalcitonin [ng/mL]	3 (0–12)	15 (5–24)	4 (1–12)	3 (0–11)	1 (0–6)	< 0.001	0.084	0.052	0.001	0.515	0.002	0.012
Interleukin 6 [pg/mL]	226 (53–648)	6882 (575–7513)	365 (103–919)	157 (60–443)	134 (28–449)	< 0.001	0.037	0.003	< 0.001	0.139	0.009	0.292
**Renal function**												
Creatinine [mg/dL]	1.3 (0.9–2.4)	1.7 (1.2–2.8)	1.3 (0.9-2.0)	1.7 (1.0-2.7)	1.1 (0.7–1.8)	0.003	0.453	0.698	0.171	0.171	0.453	0.003
Blood Urea Nitrogen [mg/dL]	27 (17–42)	35 (13–45)	25 (16–43)	34 (20–50)	23 (16–34)	0.003	0.936	0.936	0.622	0.140	0.622	0.002
Urine output [mL/24 h]	1070 (509–1848)	0 (0–39)	1000 (590–1860)	1090 (550–1750)	1300 (745–2313)	< 0.001	< 0.001	< 0.001	< 0.001	0.897	0.267	0.267
Preexisting chronic kidney disease	69 (21)	2 (13)	23 (24)	27 (27)	17 (24)	0.135	-	-	-	-	-	-
Preexisting renal replacement therapy	15 (5)	1 (7)	4 (4)	7 (7)	3 (3)	0.392	-	-	-	-	-	-
**Liver function**												
Aspartate transaminase [U/L]	58 (28–142)	3561 (1298–6071)	56 (33–142)	57 (30–120)	50 (20–87)	< 0.001	< 0.001	< 0.001	< 0.001	0.728	0.344	0.728
Alanine transaminas [U/L]	33 (18–79)	1577 (671–2477)	33 (17–59)	32 (19–76)	32 (16–69)	< 0.001	< 0.001	< 0.001	< 0.001	1.000	1.000	1.000
Total bilirubin [mg/dL]	0.6 (0.4–1.2)	1.3 (1.0-1.8)	1.0 (0.5–1.7)	0.5 (0.4–0.8)	0.5 (0.4-1.0)	< 0.001	0.264	0.002	0.002	< 0.001	0.002	0.720
**Coagulation/Hematology**												
INR	1.3 (1.2–1.6)	3.0 (2.2–3.6)	1.3 (1.2–1.7)	1.2 (1.2–1.3)	1.2 (1.1–1.3)	< 0.001	< 0.001	< 0.001	< 0.001	0.022	< 0.001	0.155
Platelets [x10^3^/µL]	197 (124–270)	59 (34–145)	203 (120–286)	192 (139–271)	203 (135–261)	0.004	0.002	0.005	0.002	1.000	1.000	1.000
Hemoglobin [g/dL]	8.7 (7.4–10.3)	7.1 (6.5–8.3)	8.5 (7.4–10.7)	8.5 (7.3–9.9)	9.5 (7.9–11.1)	0.001	0.047	0.058	0.002	0.640	0.081	0.047
Lactate dehydrogenase [U/L]	335 (235–514)	3665 (1658–4528)	313 (235–506)	364 (241–514)	303 (213–431)	< 0.001	< 0.001	< 0.001	< 0.001	0.698	0.698	0.220
**Hemodynamics/Acid Base Balance**												
Lactate [mmol/L]	2.2 (1.5–3.8)	17.0 (11.0–18.0)	2.2 (1.6–3.8)	2.0 (1.4–3.5)	2.2 (1.4–3.4)	< 0.001	< 0.001	< 0.001	< 0.001	0.653	0.653	0.978
Heart rate [/min]	85 (73–100)	100 (91–110)	91 (75–106)	84 (71–99)	78 (71–94)	0.001	0.128	0.026	0.003	0.315	0.026	0.315
Mean arterial blood pressure	81 (74–88)	73 (70–79)	81 (76–81)	79 (73–86)	82 (76–92)	0.007	0.061	0.123	0.008	0.417	0.399	0.123
Norepinephrine [mg/h], maximum dose	1.0 (0.2-4.0)	7.0 (5.2–12.0)	1.5 (0.4–4.5)	0.6 (0.0-3.4)	0.8 (0.0-2.4)	< 0.001	0.004	< 0.001	< 0.001	0.098	0.029	0.525
pH	7.3 (7.3–7.4)	7.1 (7.1–7.2)	7.3 (7.3–7.4))	7.3 (7.3–7.4)	7.4 (7.3–7.4)	< 0.001	< 0.001	< 0.001	< 0.001	0.784	0.192	0.208
Base excess [mmol/L]	0.1 (−3.1-2.7)	−6.9 (−10.3-(−5.7))	0.3 (−3.2-2.7)	−0.5 (−2.6-2.1)	0.7 (−1.7-3.2)	< 0.001	< 0.001	< 0.001	< 0.001	0.954	0.483	0.483
**Respiration**												
Horowitz index [mmHg]	233 (157–295)	233 (215–282)	237 (159–323)	240 (171–292)	211 (131–273)	0.336	-	-	-	-	-	-
Mechanical ventilation	233 (84)	14 (100)	67 (83)	76 (83)	76 (83)	0.414	-	-	-	-	-	-

Numeric values are represented as median and interquartile range (IQR), categorical values as absolute and relative frequencies. All values were obtained on first day of sepsis. p-values were calculated by Chi-square test for categorical data and Kruskal-Wallis test for continuous data. The reported relative frequencies are based on the actual number of available data points for each variable. Post-hoc p values were corrected for multiple testing by Bonferroni method

^1^ Survival time for patients who died within 30 days

CNS: Central nervous system. COVID-19: Coronavirus disease 2019. SOFA: Sequential organ failure assessment. WBC: White blood cells. INR: International normalized ratio. mmHg: Millimeter mercury

### Immune status

At day 1, we found significant differences in the levels of IL-6, IL-8, IL10, IL-18 and CCL-2 (Supplementary Table 4). The distribution of cytokine concentrations resembled the distribution of the SOFA score, with the highest levels observed in cluster 0 and lowest in cluster 3 (Fig. 1e). At day 4, we additionally found IL-17a to be significantly differential between clusters 1 and 3. The counts of lymphocytes and white blood cells were not significantly differential between the subtypes.

### Characteristics of the sepsis plasma proteome subtypes

**Fig. 2:**
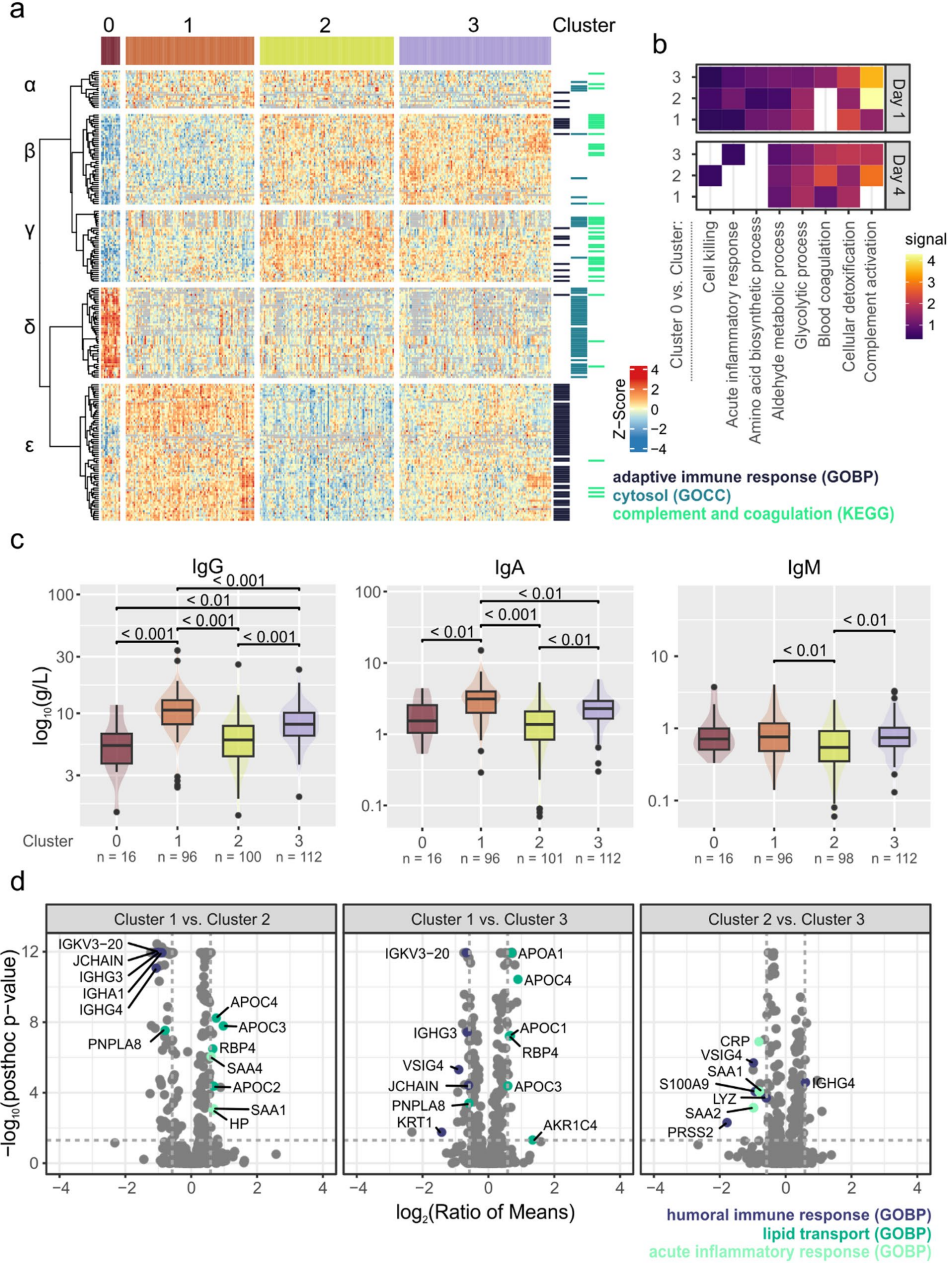
Proteome characteristics of sepsis plasma proteome subtypes. **a** Heatmap showing significantly differentially abundant proteins at day 1 (ANOVA pFDR value ≤ 0.05, post-hoc test p value ≤ 0.05, ratio of means ≥ 1.5 or ≤ 0.67). Protein intensities were z-transformed and clustered using Pearson clustering with Ward’s linkage method. The first five branches in the dendrogram were divided and labeled with Greek letters to discriminate abundance patterns. Protein annotation with selected Gene Ontology or KEGG categories is indicated on the right. **b** Functional enrichment of significantly differential proteins in comparison to cluster 0. Eight selected categories shown for day 1 and day 4 and the three pairwise comparisons with cluster 0. Enrichment analysis was done with string-db.org (v.12) using GO biological processes (GOBP). **c** Immunoglobulin measurements as boxplot representation. Boxes represent 25th and 75th percentiles, whiskers extend to the most extreme data points, median shown as a horizontal line, outliers shown as individual data points, p-values calculated by Dunn’s post-hoc test. **d** Volcano plots illustrating the pairwise comparisons between clusters 1, 2 and 3 for day 1. Proteins annotated with significantly enriched GOBP terms were highlighted and labeled with gene names. Dashed lines indicate the applied significance threshold

Proteome analysis revealed the greatest differences between the subtypes in comparison to cluster 0. Overall, we found 271 significantly differential proteins between all four clusters at day 1 and 206 proteins at day 4 (Fig. 2a, Supplementary Fig. 11, Supplementary Table 8). Of these, 247 and 181 proteins were differential in comparison to cluster 0 at day 1 and day 4, respectively. This illustrates the unique plasma composition of cluster 0 which was associated with the most severe sepsis and highest mortality. The hierarchical cluster analysis of all significant proteins revealed five characteristic abundance patterns in relation to the identified subtypes on day 1. On day 4, four patterns could be discriminated, with regulation pattern α missing (Supplementary Figs. 11 and 12, Supplementary Table 9).

Typical of cluster 0, we found markers of cell death and tissue damage to be elevated, characterized by their common cytosolic location (Fig. 2a, abundance pattern δ). These consisted mainly of liver proteins associated with metabolic processes such as glucose, aldehyde and amino acid metabolism as well a cellular detoxification (Fig. 2b). The enriched biological processes might indicate an accumulation of the corresponding proteins before cell death, pointing towards enhanced elimination of reactive oxygen species and a metabolic shift towards anaerobic glycolysis. On the other hand, we found proteins related to blood coagulation but also complement activation and acute phase response to be depleted in cluster 0, showing the lowest levels across all subtypes (abundance patterns β and γ).

Decreasing levels of proteins associated with complement and coagulation as well as acute phase response were also apparent in cluster 1, highlighting the relevance of the associated processes in sepsis. Similarly, apolipoproteins, especially related to remodeling of high-density lipoprotein particles, were lower abundant in cluster 1 compared to clusters 2 and 3 (Fig. 2d, Supplementary Fig. 11b and c), showing even more pronounced depletion in cluster 0 (abundance pattern β). This finding was in accordance with clinical values, which indicated reduced liver function in cluster 1 and acute liver failure in cluster 0 (Fig. 1d). Another striking finding was the elevation of immunoglobulins (Ig) in cluster 1, in particular because this observation could not be anticipated based on clinical routine data (Fig. 2a, abundance pattern ε). High levels of immunoglobulins indicated a strong adaptive immune response in this subtype, corresponding to high SOFA scores and cytokine levels. The lowest immunoglobulin levels, in contrast, were found in cluster 2 which also corresponds to characteristically low levels in total protein. As no clinical routine parameter could be used to specifically substantiate these observations, total levels of IgG, IgA and IgM were determined post-hoc. These independent measurements verified the complementary levels of IgG and IgA, and showed the lowest levels of IgM in cluster 2 (Fig. 2c). In cluster 2, however, the highest levels of proteins related to acute inflammatory response were observed, including Serum amyloid A1 and A2 (SAA1, SAA2) as well as Haptoglobin (HP), and on day 4 also CRP (Fig. 2a, abundance pattern γ, Fig. 2d). Within the clusters 1, 2 and 3, the highest levels of the alarmins S100 A8 and A9 were also found in cluster 2 (Supplementary Fig. 11).

On day 1, an additional regulation pattern of proteins was observed, showing their lowest levels in cluster 3 (Fig. 2a, α pattern). This set of proteins contained proteins related to leukocyte transmigration (ICAM1, VCAM1), the kidney function marker Cystatin C (CST3) and immunoregulatory proteins such as VSIG4. A similar regulation pattern was not observed on day 4. The lowest abundance in cluster 3 as well as the increasing levels corresponding with increasing disease severity underlined the baseline character of cluster 3.

### Prediction of sepsis plasma proteome subtypes by supervised machine learning

**Fig. 3:**
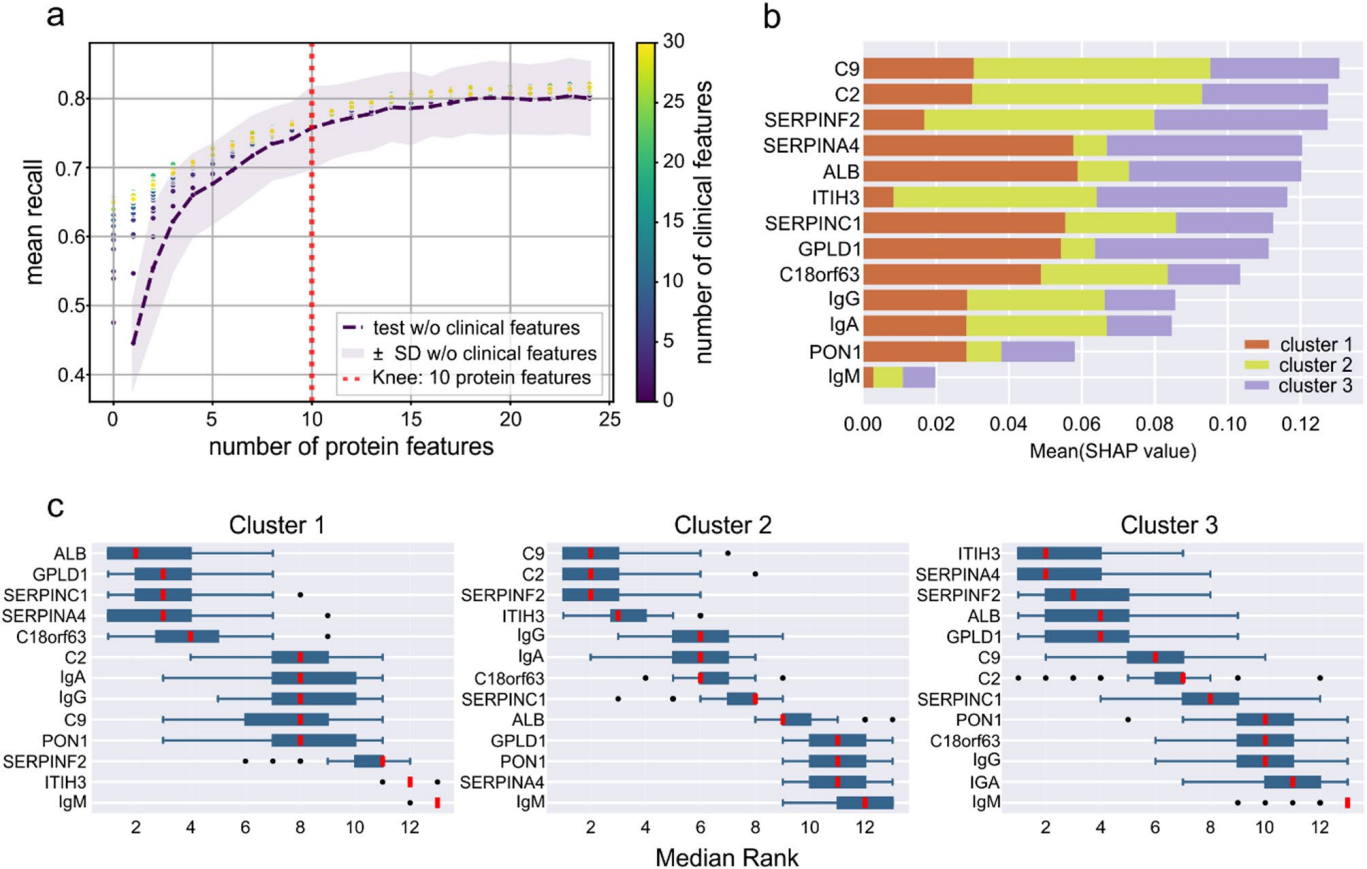
Feature selection and machine learning model performance: **a** Scatter plots representing the mean recall as a function of the number of medical features included. The purple dashed line shows the mean recall when using only protein features. The transparent area curve represents the standard deviation. The red dotted line indicates the computed knee point, where the slope of the curve decreased significantly. With only few proteins available, medical features had a stronger impact on model performance, with increasing number of proteins this effect diminished. **b** Bar plot showing the mean SHAP values for the features used in model training. The colours indicate the contribution of each feature to the respective clusters. **c** Box plots of feature ranks for each cluster, based on the relevance of the features within the model across all MCCV iterations. Boxes represent 25th and 75th percentiles, whiskers extend to the most extreme data points, median shown as a red line, outliers shown as individual data points

A random forest machine learning (ML) classifier was generated to showcase patient stratification according to sepsis plasma proteome subtypes. First, the number of features beyond which only a marginal improvement in recall was achieved was identified by iteratively adding features to the model and evaluating recall Classifiers based mainly on clinical features showed only poor performance, highlighting the complementarity of clinical routine measurements and plasma proteomics data (Fig. 3a, Supplementary Table 10). Over 100 iterations of Monte Carlo cross-validation (MCCV), the most frequently selected proteins in combinations with ten features were encoded by the following genes: C9, ITIH3, ALB, GPLD1, C2, SERPINF2, C18orf63, SERPINC1, SERPINA4, and PON1 (Table 2, Supplementary Fig. 13). At this level, the inclusion of clinical features resulted in only minimal performance gains. We then used the identified features to train a new model and evaluate its metrics by MCCV. As Igs provided particularly relevant information for differentiation of clusters 1 and 2, they were also incorporated into the final model. Here, a discrepancy greater than 0.1 was observed between the training and test sets for all evaluated metrics, indicating overfitting, likely caused by an insufficient number of data points. Despite overfitting, an acceptable model performance was still achieved for test data across all three clusters with an AUROC of 0.932 ± 0.022 as well as a precision of 0.703 ± 0.066 and a recall of 0.802 ± 0.044 (Table 3). We assessed the contribution of individual features to the model predictions and found C9, C2 and SERPINF2 (Fig. 3b) with the overall highest predictive values. In Cluster 1, the most important features were ALB, GPLD1, SERPINC1 and SERPINA4 (Fig. 3c), which were associated with lower values, while C18orf63 levels were elevated (Supplementary Fig. 14). In Cluster 2 the most important features C9, C2, SERPINF2 and ITIH3 showed increased levels. In Cluster 3, ITIH3 and SERPINA4 showed the highest importance, with lower levels of ITIH3 and higher levels of SERPINA4 being associated with cluster-specific predictions.

## Discussion

The identified plasma proteome subtypes were characterized by different sepsis severity as reflected by the SOFA score and cytokine quantities. Cluster 0 represented the most severe patients with shortest survival and 100% mortality. This was accompanied by clinical routine data which showed overall extreme values, pointing towards liver failure, acute kidney injury and DIC. Based on these features, cluster 0 seemed to represent rather a terminal stage in sepsis progression than a molecular subtype in the sense of a disease endotype. A significantly shorter time of ICU and hospital stay for patients in cluster 0, however, indicated a rapid worsening that was characteristic to this subtype (Supplementary Fig. 8). We conclude that a finer temporal resolution might clarify which patients migrate through cluster 0 when dying, and if it represents an endotype, a time point in sepsis or a mixture of both.

**Table 2:** Gene and proteins names of the selected biomarker panel

Gene	UniProt ID	Protein name
ALB	P02768	Albumin
C18orf63	Q68DL7	Uncharacterized protein C18orf63
C2	P06681	Complement C2
C9	P02748	Complement component C9
GPLD1	P80108	Phosphatidylinositol-glycan-specific phospholipase D
ITIH3	Q06033	Inter-alpha-trypsin inhibitor heavy chain H3
PON1	P27169	Serum paraoxonase/arylesterase 1
SERPINA4	P29622	Kallistatin
SERPINC1	P01008	Antithrombin-III
SERPINF2	P08697	Alpha-2-antiplasmin

For the other three clusters, we observed striking evidence of different immune responses, with clusters 1 and 2 showing opposite phenotypes. Cluster 1 was characterized by a comprehensive immune reaction, including the adaptive immune system as indicated by high Ig levels. Decreasing complement and coagulation factors as well as acute phase proteins also suggested activation of the innate immune system and consumption of the corresponding proteins. Cluster 2, in contrast, showed the lowest Ig levels but highest levels of acute phase proteins. In addition, elevated intensities of S100A8/A9 proteins hinted towards a pronounced neutrophil degranulation. The immunomodulatory protein VSIG4 indicated a monocytic immune response and activation of macrophages [19]. Complement and coagulation factors remained on baseline level, as inferred from the mildest subtype 3, which was interpreted as the sepsis plasma proteome baseline of the patients in the investigated cohort. This assumption was supported for example by VCAM1 and ICAM1, which at day 1 showed the lowest levels in cluster 3 (regulation pattern α), and for which increasing plasma levels are well known to be related to sepsis severity [20].

**Table 3:** Performance metric of the random forest ML classifier. The classifier was based on 10 proteins and additional Ig quantities and evaluated using 100 times Monte Carlo cross validation

	Test Set		Training Set	
Metric	Mean	SD	Mean	SD
Accuracy	0.800	0.044	0.941	0.009
AUROC	0.932	0.022	0.994	0.001
F1 Score	0.801	0.044	0.942	0.009
MCC	0.801	0.044	0.942	0.009
Precision	0.703	0.066	0.912	0.014
Recall	0.802	0.044	0.941	0.009

Although we found SOFA scores to be gradually decreasing between clusters 1, 2 and 3, no significant difference in 30-day mortality was observed at day 1. Contrary to these findings, the ALBIOS trial reported higher mortality after 90 days to be associated with increased IgG and IgA levels [21]. In addition, a decrease of high-density lipoproteins has been reported to be predictive for sepsis mortality [22]. Both observations match the increased SOFA scores and cytokine levels in cluster 1, which themselves are predictive for higher mortality [23], so a worse outcome for patients in cluster 1 might have been anticipated. Unfortunately, a later endpoint such as 90-day survival and adverse events like secondary infections, were not assessed in our study.

Sepsis subtypes, also termed subclasses, phenotypes or endotypes, often used synonymously [24], are considered a major way towards personalized therapy in sepsis. In contrast to clinical subtypes which rely mostly on clinical routine and laboratory data [13, 25], molecular subtypes have the potential to provide direct insights into pathophysiology. Until now, multiple molecular subtypes have been reported and were often based on transcriptome analysis using whole blood or immune cells [14, 26–28], but also multiplex cytokine measurements or the combination of both [29, 30]. Recently, the first study describing three molecular subtypes based on plasma proteomics data has been published [10]. Of note, Mi and colleagues also observed a subtype which was characterized by high Ig levels and low levels of apolipoproteins as well complement activation (termed SPC1). The overlap with our observations implies generalizability of this subtype, and highlights its potential clinical relevance, for example in patient stratification in clinical trials. One therapeutic consideration that obviously may be influenced by plasma proteome subtypes is the use of intravenous (IgM-enriched) immunoglobulin preparations, which have shown heterogenous results in clinical trials and are still under debate [31, 32].

While sepsis plasma proteome subtypes provide clinically relevant information, the assignment of new patients to the subtypes remains challenging. As mass spectrometry-based plasma proteomics are currently unlikely to be implemented into clinical routine, we generated a ML classifier based on only 10 proteins. Additional Ig quantities were measured on a routine diagnostics platform and assays for albumin and antithrombin III (SERPINC1) are readily available. In future, the remaining proteins could be measured using immunoassays, ideally in a multiplex manner. The classifier differentiates only subtypes 1–3, as cluster 0 was too small to generate a robust ML model. However, patients with most severe sepsis might be identified based on clinical routine data as described previously [33]. The most important protein for differentiation of plasma subtypes 1–3 was Complement C9 (C9). C9 is a component of the membrane attack complex (MAC) and low levels of C9 were found to be associated with septic shock in patients with Gam-negative bacteremia [34]. Complement factor C2, part of the classical pathway, was found to be the second most important protein, underlining the relevance of the complement system in sepsis. Another highly relevant feature was serum albumin (ALB). Interestingly, low levels of albumin, as indicative for cluster 1, were previously reported to be associated with poor prognosis [35, 36], which supports the impression that cluster 1 might be associated with a worse outcome than observed using the 30-day survival endpoint. In addition, a decrease of the kallikrein inhibitor kallistatin (SERPINA4), also indicative for cluster 1, was reported to be associated with septic shock and sepsis mortality [37]. Antithrombin III (SERPINC1) as well as Alpha-2-antiplasmin (SERPINF2) are both important regulators of the coagulation cascade and were reported to be associated with sepsis mortality and organ dysfunction [7]. The alpha-trypsin inhibitor ITIH3 has been described in sepsis [38] as well as to be related to mortality in severe COVID-19 [39] and the activity of the serum paraoxonase PON1 has been shown to be predictive for 30-day mortality [40]. Taken together, there is ample evidence for the diagnostic relevance of most of the selected proteins, underlining the value of the model and suggesting that of our findings might be applicable in independent cohorts. The developed model illustrates that the transfer between different technological platforms is not necessary an obstacle to transferring proteomics subtypes into the clinics and opens up a perspective on how patient stratification could be realized.

### Limitations

The major limitation of our study is its limited cohort size, which makes it necessary to verify our findings in independent cohorts to evaluate their generalizability. This should especially be considered for the small cluster 0, which however had a great impact on the proteome characteristics of our data set due to the moribund nature of the respective patients. Consequently, the developed machine learning model and biomarker panel represent a proof-of-concept how proteomics results might translate to the clinics and require testing in independent cohorts. We assume that the observed sepsis plasma proteome subtypes show features that will be reproducible in other cohorts, a hypothesis that is strengthened by agreements with the observations of Mi et al. [10]. Our study is also limited regarding the evaluation of endpoints other than 30-day survival. Differences in longer survival times or secondary infection cannot be assessed. We did not examine the influence of therapeutic measures on the plasma proteome subtypes due to the lack of appropriate data. The presented analyses are limited with respect to plasma proteome coverage. Due to limitations in instrumentation and the specific protein composition of plasma, it is currently extremely challenging to measure low-abundance proteins, such as certain cytokines, using LC-MS/MS. Consequently, our observations are limited to the top 600 plasma proteins, and we cannot make statements regarding lower abundant proteins.

## Conclusions

The identified sepsis plasma proteome subtypes are characterized by distinct features that provide insights into the underlying disease mechanisms and immune processes of this heterogeneous syndrome. These findings allow conclusions on benefits and disadvantages of therapeutic interventions, and have the potential to support the predictive enrichment of patients in clinical trials. In a clinical setting, patient stratification could be achieved by measuring a representative biomarker panel. These findings represent a significant advancement towards precision medicine in sepsis management.

## Supplementary Information

Supplementary Material 1

Supplementary Material 2

Supplementary Material 3

Supplementary Material 4

Supplementary Material 5

## Data Availability

The mass spectrometry proteomics data have been deposited to the ProteomeXchange Consortium via the PRIDE partner repository with the dataset identifier PXD064125.

## Abbreviations

AKI: Acute kidney injury
ALT: Alanine transaminase
AST: Aspartate transaminase
AP: Alkaline phosphatase
aPTT: Activated partial thromboplastin time
AUROC: Area under the receiver operating characteristic curve
CHE: Cholinesterase
CNS: Central nervous system
CRP: C-reactive protein
DIC: Disseminated intravascular coagulation
FDR: False discovery rate
GO: Gene ontology
ICU: Intensive care unit
Ig: Immunoglobulin
INR: International normalized ratio
IQR: Interquartile range
LDH: Lactate dehydrogenase
LOD: Limit of detection
MCCV: Monte carlo cross-validation
ML: Machine learning
PCA: Principal component analysis
SHAP: SHapley additive exPlanations
SOFA: Sepsis-related organ failure assessment score
